# Association between pan-immune-inflammation value and coronary heart disease in elderly population: a cross-sectional study

**DOI:** 10.3389/fcvm.2025.1538643

**Published:** 2025-02-10

**Authors:** Ruicong Ma, Jinyi Ren, Xianmei Chen, Xia Li, Ying Zhao, Yanchun Ding

**Affiliations:** ^1^ Department of Cardiology, The Second Hospital of Dalian Medical University, Dalian, Liaoning, China; ^2^ Department of Immunology, College of Basic Medical Science, Dalian Medical University, Dalian, Liaoning, China; ^3^ Molecular Medical Laboratory, College of Basic Medical Science, Dalian Medical University, Dalian, China

**Keywords:** coronary heart disease, NHANES, pan-immune-inflammation value, controlling nutritional status score, elderly people

## Abstract

**Background:**

Systemic inflammation, immune and nutrition status are closely linked to the occurrence and development of coronary heart disease (CHD). Pan-immune-inflammation value (PIV) is a new method for evaluating systemic inflammation and immune status. Our objective is to explore the connection between PIV and CHD especially in elderly people, as well as the diagnostic value of PIV combined with controlling nutritional status (COUNT) score for CHD.

**Methods:**

Participants eligible for the study were sourced from NHANES data from 1999 to 2018. Logistic regression models were employed to evaluate the link between PIV and CHD. Additionally, restricted cubic spline was utilized to explore the correlations. Subgroup analysis was adopted in order to ensure the credibility of the results. The receiver operator characteristic (ROC) curve was used to explore the predictive value of PIV combined with COUNT score for CHD.

**Results:**

41,713 individuals qualified for analysis. The individuals with CHD had higher levels of PIV. In the logistic regression model, PIV was positively related to CHD [Q4 vs. Q1, OR = 1.23 (1.03–1.48, *P* < 0.001)]. Restricted cubic spline indicated a positive non-linear relationship (*P* for overall <0.001, *P* for non-linear = 0.009). However, restricted cubic spline shows that this positive correlation is only significant in the elderly population aged 60 and above. Subgroup analysis shows that the relationship between PIV and CHD is more significant in the elderly population (*P* < 0.001). The ROC curve shows that PIV has better diagnostic value for CHD than other common inflammatory indicators. Furthermore, the combination of PIV and COUNT score is superior to PIV or COUNT score.

**Conclusions:**

A positive link between PIV and CHD, especially in the elderly. The combination of PIV and COUNT score has better diagnostic value for CHD.

## Introduction

Cardiovascular diseases (CVDs) rank as the foremost cause of mortality across the globe, representing approximately one-third of all annual fatalities (1, 2). Coronary heart disease (CHD) is the main type of CVDs and the leading cause of cardiovascular mortality in both men and women (3, 4). In addition, the incidence of CHD is increasing year by year with the intensification of population aging. The development of CHD can also cause serious adverse cardiovascular events, including myocardial infarction, heart failure, malignant arrhythmia, etc. (5). Research shows that more than 20 million Americans suffer from CHD, which brings huge economic and medical burden to the country (6, 7).

The pathological basis of CHD is atherosclerosis.Atherosclerosis is a chronic inflammatory disease driven by lipid. Chronic inflammation can induce endothelial cells to release adhesion molecules, promote smooth muscle cell proliferation and migration, and exacerbate the infiltration of pro-inflammatory immune cells. These pathological processes can accelerate the progression of atherosclerosis (8, 9). The development of CHD is significantly influenced by inflammation and the immune response. These factors are intricately connected. Inflammation can promote immune response, leading to further inflammation in turn. Under the constant stimulation of immune response and inflammatory effect, atheromatous plaque can gradually increase, inducing plaque rupture and thrombosis, which ultimately leads to adverse cardiovascular events. Although many measures have been taken, the prevention and treatment of atherosclerosis are still difficult. Therefore, further development of biomarkers for predicting the risk of disease occurrence and improving patient risk stratification is crucial for the general public.

Common indicators of blood routine include neutrophils, lymphocytes, monocytes, platelets, etc. Researchers have found that the combination of these indicators can better reflect the inflammatory state of the body and is closely related to the increased risk of diseases (10, 11). In addition, pan-immune inflammation value (PIV), a novel marker of inflammation, was developed for research, integrating four types of immune cells including neutrophils, monocytes, platelets, and lymphocytes. The definition of PIV is as follows: neutrophil count × platelet count × monocyte count/lymphocyte count (12). Due to its potential ability to comprehensively reveal the status of systemic immune and cancer-related inflammatory responses, PIV is considered a reliable predictor of clinical outcomes in patients with inflammatory diseases and cancer (13). - In contrast to other immune indices such as neutrophil-to-lymphocyte ratio (NLR), monocyte-to-lymphocyte ratio (MLR) and systemic immune inflammation index(SII), PIV seems to have higher predictive value for diseases (14–16).

In addition, patients with CHD are often elderly and suffer from malnutrition (17, 18). There is also a close relationship between inflammation and malnutrition. The relationship between inflammation and malnutrition may be bidirectional. Inflammation is a driving factor for malnutrition, and malnutrition can exacerbate inflammation (19). The controlling nutritional status (COUNT) score is a classic evaluation of the body's nutritional status by taking into account the composition of lymphocytes, levels of total cholesterol, and albumin (20). It involves three aspects of protein reserves, calorie expenditure, and immune defense in the body. Furthermore, it can be used as an early screening tool for malnutrition in hospitalized patients and continuous monitoring of nutritional status during treatment (21). PIV is used to measure the level of inflammation in the body, while COUNT score is used to measure the nutritional status of the body. The difference between the two is significant. Therefore, we also want to further explore whether the combination of PIV and COUNT score has better predictive value for CHD.

PIV is associated with many diseases, including tumors, chronic obstructive pulmonary disease, and hypertension (22–24). Nonetheless, the correlation between PIV and CHD risk remains ambiguous. Consequently, the objective is to explore the link between PIV and CHD, as well as the diagnostic value of PIV combined with controlling nutritional status (COUNT) score for CHD.

## Materials and methods

### Data source and sample selection

**Figure 1:**
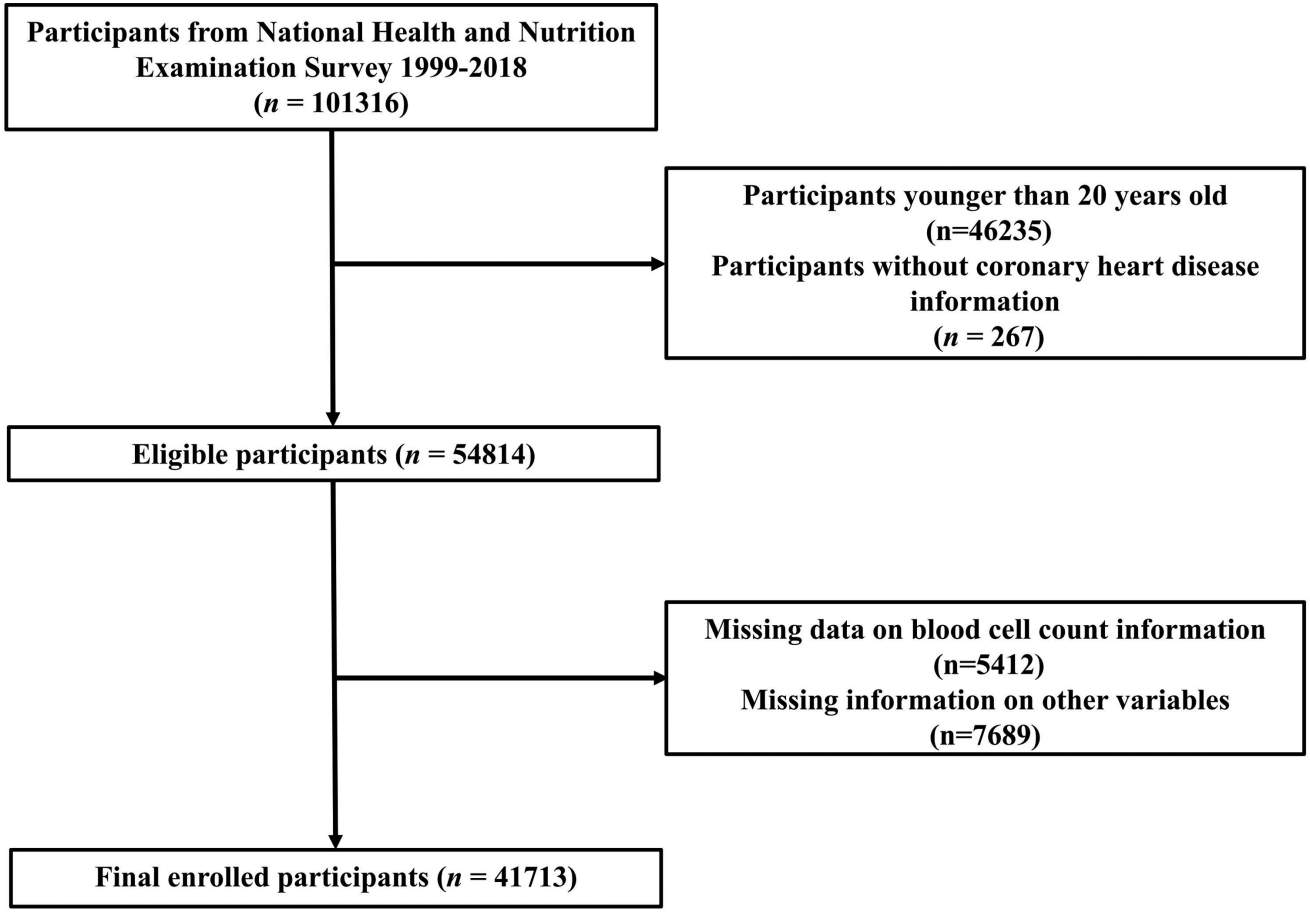
The flow chart of participant selection.

Data for this study were obtained from the National Health and Nutrition Examination Survey (NHANES) database (https://www.cdc.gov/nchs/nhanes/). We aim to include adults with complete information from the NHANES database from 1999 to 2018. Initially, a total of 101,316 participants were included in the database from 1999 to 2018. First, we excluded individuals under 20 years of age (*n* = 46,235) and those without CHD data (*n* = 267). The calculation of PIV and COUNT scores requires complete hematological examination results, and we further excluded indiiduals with missing incomplete blood cell information (*n* = 5,412). Due to the possible impact of missing covariates on the results, we also excluded participants who were missing data on various covariates (*n* = 7,689). As a result, the final sample for analysis included 41,713 participants. The details of the screening methodology are illustrated in Figure 1.

### Calculation of inflammatory indicators and COUNT score

PIV is calculated by integrating four types of immune cells, including neutrophils, monocytes, platelets, and lymphocytes. The calculation formula for PIV, NLR, MLR and SII are as follows:

PIV = neutrophil count × platelet count × monocyte count/lymphocyte count (25). NLR = neutrophil count/lymphocyte count (26). MLR = monocyte count/lymphocyte count (27). SII = neutrophil count × platelet count/lymphocyte count. The CONUT score acts as a measure for assessing nutritional and immune status by taking into account the composition of lymphocytes, levels of total cholesterol, and albumin (28). The COUNT score is the sum of the scores corresponding to three indicators. The specific scoring criteria are shown in Supplementary Table S2.

### Covariates

Based on previous research on CHD in NHANES, we also obtained other variables from the database, including age, sex, race, body mass index (BMI), education level, blood lipids, uric acid, glomerular filtration rate (eGFR), and glycated hemoglobin (HbA1c). In addition, we also extracted smoking, drinking, hypertension and diabetes mellitus (DM).

### Statistical analysis

Initially, the dataset was categorized into two distinct groups: the non-CHD group and the CHD group. Continuous variables were expressed as mean ± SEM (Standard Error of the Mean), while categorical variables were represented as proportions (95% CI), with *P* values <0.05 considered statistically significant. Second, we conducted weighted logistic regression analysis. This statistical method was employed to investigate the potential link between PIV and CHD. Two models were developed: Model I, and Model II. Model I was adjusted for age, gender, and race. Model II was adjusted for age, sex, race, BMI, education attainment, total cholesterol(TC), high-density lipoprotein cholesterol (HDL-C), smoking, alcohol consumption, uric acid, eGFR, hypertension and DM. Restrictive cubic spline is commonly used to model the relationship between continuous variables and dependent variables. Especially in regression analysis, it allows for capturing non-linear relationships and avoiding overfitting while maintaining smoothness. Nonlinear is closer to the essence of objective things and is one of the important methods for quantitatively studying and understanding complex problems. Restricted cubic spline was utilized to examine the link between PIV and CHD. NHANES data comes from representative populations in various parts of the United States, and weighted analysis can accurately reflect the characteristics of the overall population. In addition, some studies have indicated that outcomes from weighted analyses and unweighted analyses may occasionally differ. To verify our findings, we conducted unweighted logistic regression. Furthermore, subgroup analysis was carried out as well. The receiver operator characteristic (ROC) curve is used to compare the superiority and inferiority of different inflammatory indicators in predicting CHD. Due to the close relationship between inflammation and malnutrition, we also investigated the predictive value of PIV combined with COUNT score for CHD.

## Results

### The baseline characteristics of participants

41,713 adults were screened for this study. Table 1 presents the clinical characteristics of all participants, encompassing a variety of clinical details. Firstly, we divided the patients into two groups: the CHD group and the non-CHD group. Table 1 illustrates that the participants in the CHD group were of an older age. In contrast, those in the non-CHD group displayed a lower prevalence of smoking (45.9%), diabetes mellitus (11.73%) and hypertension (35.71%). The incidence of obesity is higher in the CHD group, and the levels of uric acid and HbA1c are also higher. On the contrary, patients with CHD have lower HDL-C and are accompanied by a decline in renal function (*P* < 0.001).As expected, patients with CHD have higher COUNT scores (*P* < 0.001). This also indicates that patients with CHD are to some extent accompanied by malnutrition. It is worth noting that we also observed a significant statistical difference in lnPIV between the two groups (non-CHD vs. CHD: 5.56 ± 0.01 vs. 5.71 ± 0.02, *P* < 0.001***). Supplementary Table S1 presents clinical data based on PIV quartiles. The incidence of CHD gradually increased with the increase of PIV (*P* < 0.001). Compared with the highest PIV group, patients in the lowest PIV group had lowest CHD incidence. With the increase of PIV, the proportion of smoking, hypertension and diabetes gradually increases (*P* < 0.001). Furthermore, notable variations were also observed in terms of age, ethnicity, BMI, HbA1c, eGFR, HDL-C, TC, uric acid, alcohol consumption and educational attainment between the four groups (*P* < 0.001).

**Table 1:** Clinical characteristics of population.

Variables	Overall	Non-CHD	CHD	*P* value
Age, %	47.21 ± 0.19	46.50 ± 0.18	66.59 ± 0.34	<0.001
Sex, %				<0.001
Female	50.54 (48.64, 52.44)	51.11 (50.60, 51.63)	34.75 (31.71, 37.80)	
Male	49.46 (47.66, 51.27)	48.89 (48.37, 49.40)	65.25 (62.20, 68.29)	
Race/ethnicity, %				<0.001
White	70.05 (65.84, 74.27)	69.58 (67.57, 71.59)	83.10 (80.87, 85.34)	
Black	10.22 (9.34,11.11)	10.38 (9.31, 11.45)	5.81 (4.85, 6.77)	
Mexican	8.01 (7.04, 8.98)	8.18 (7.10, 9.26)	3.39 (2.42, 4.35)	
Others	11.71 (10.75, 12.67)	11.86 (10.81, 12.91)	7.71 (5.98, 9.43)	
Education levels, %				<0.001
Less than high school	16.49 (15.52, 17.45)	16.21 (15.32, 17.10)	24.08 (21.46, 26.71)	
High school or equivalent	23.98 (22.66, 25.29)	23.90 (23.08, 24.73)	25.99 (22.88, 29.09)	
College or above	59.54 (57.01, 62.06)	59.89 (58.52, 61.25)	49.93 (46.47, 53.39)	
BMI, kg/m2	28.77 ± 0.07	28.73 ± 0.07	29.93 ± 0.19	0.001
InPIV	5.56 ± 0.01	5.56 ± 0.01	5.71 ± 0.02	<0.001
HbA1c, %	5.57 ± 0.01	5.55 ± 0.01	6.14 ± 0.04	<0.001
TC, mmol/L	5.08 ± 0.01	5.09 ± 0.01	4.64 ± 0.04	<0.001
HDL, mmol/L	1.37 ± 0.00	1.38 ± 0.00	1.26 ± 0.01	<0.001
Uric acid, umol/L	322.85 ± 0.63	321.46 ± 0.64	361.19 ± 3.00	<0.001
eGFR, mL/min/1.73 m^2^	93.79 ± 0.26	94.59 ± 0.25	71.94 ± 0.62	<0.001
COUNT score	0.70 ± 0.01	0.68 ± 0.01	1.22 ± 0.04	<0.001
Smoking, %				<0.001
No	53.42 (51.50, 55.35)	54.10 (53.14, 55.06)	34.86 (31.90, 37.82)	
Yes	46.58 (44.47, 48.68)	45.90 (44.94, 46.86)	65.14 (62.18, 68.10)	
Drinking, %				<0.001
No	25.60 (23.90, 27.30)	25.09 (23.78, 26.39)	39.77 (36.50, 43.04)	
Yes	74.40 (71.60, 77.20)	74.91 (73.61, 76.22)	60.23 (56.96, 63.50)	
Hypertension, %				<0.001
No	62.90 (60.57, 65.24)	64.29 (63.47, 65.11)	24.73 (21.98, 27.48)	
Yes	37.10 (35.47, 38.72)	35.71 (34.89, 36.53)	75.27 (72.52, 78.02)	
DM, %				<0.001
No	87.30 (84.13, 90.47)	88.27 (87.78, 88.76)	60.70 (57.98, 63.42)	
Yes	12.70 (12.04, 13.35)	11.73(11.24, 12.22)	39.30(36.58, 42.02)	

Continuous data were presented as the mea*n* ± SEM, category data were presented as the proportion and 95% confidence interval. SEM, standard error of the mean; PIV, pan-immune-inflammation value; BMI, body mass index; HbA1c, glycosylated hemoglobin; TC, total cholesterol; HDL-C, high-density lipoprotein cholesterol; eGFR estimated glomerular fltration rate; DM, diabetes mellitus; CHD, coronary heart disease.

### The link between PIV and CHD

As a continuous variable, a positive correlation was showed between PIV and CHD, with an OR of 1.42 (95%CI: 1.28–1.56) in unadjusted logistic regression analysis (Table 2). After adjusting the confounding factors in Model II, this relationship still exists(OR = 1.12, 95%CI: 1.01–1.24). As a categorical variable, the highest quartile also showed a highest risk of CHD in Model II after adjusting the confounding factors (OR = 1.23, 95%CI: 1.03–1.48).

**Table 2:** Weighted logistic regression analysis on the correlation between PIV and CHD.

	Non-adjusted model		Model I		Model II	
	OR [95% CI]	*P* value	OR [95% CI]	*P* value	OR [95% CI]	*P* value
Continuous PIV	1.42 (1.28, 1.56)	<0.001***	1.20 (1.09, 1.33)	<0.001***	1.12 (1.01, 1.24)	0.03*
Q1	Reference	–	Reference	–	Reference	–
Q2	1.27 (1.04, 1.55)	0.02*	1.17 (0.95, 1.45)	0.13	1.15 (0.94, 1.41)	0.19
Q3	1.39 (1.14, 1.71)	0.001**	1.21 (0.99, 1.48)	0.06	1.18 (0.96, 1.46)	0.11
Q4	1.80 (1.51, 2.15)	<0.001***	1.37 (1.15, 1.64)	<0.001***	1.23 (1.03, 1.48)	0.02*

Data are presented as OR (95% CI). PIV, pan-immune-inflammation value; CHD, coronary heart disease; Model I adjusted for age, sex and race/ethnicity. Model II adjusted for age, sex, race/ethnicity, education levels, BMI, smoking, drinking, TC, HDL-C, uric acid, eGFR, hypertension and DM.

* *P* value <0.05.

** *P* value <0.01.

*** *P* value <0.001.

### Restricted cubic spline

**Figure 2:**
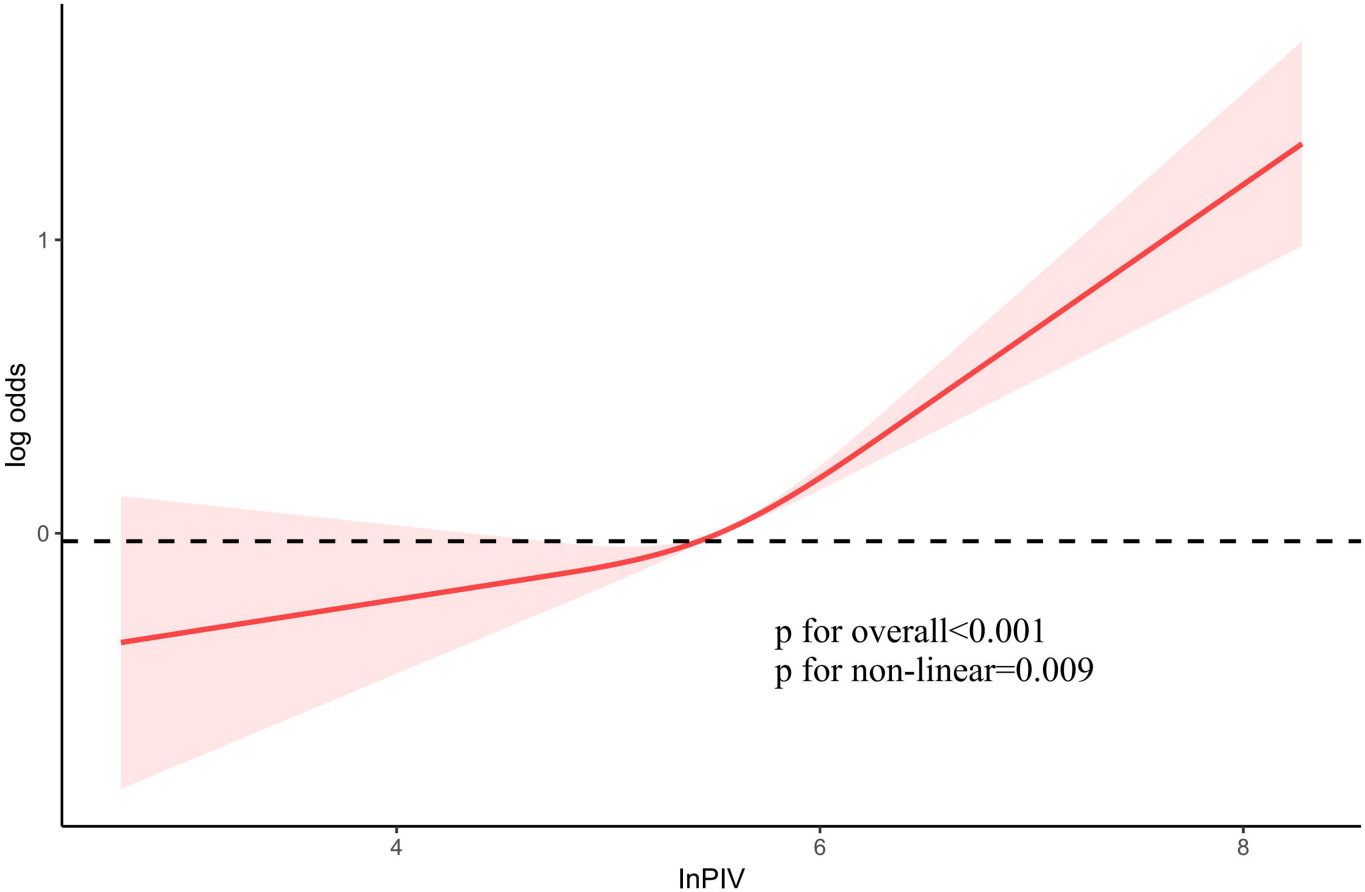
The link between PIV and the risk of CHD. PIV, pan-immune inflammation value; CHD, coronary heart disease.

**Figure 3:**
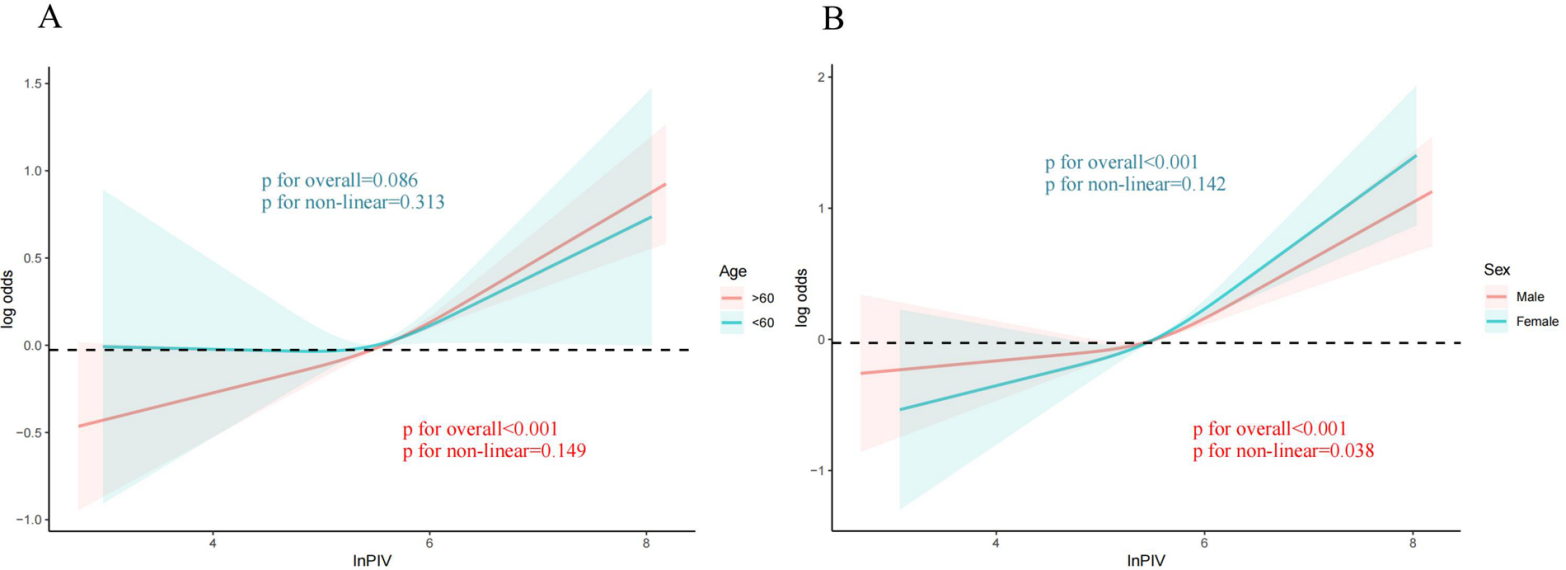
The link between PIV and CHD in different subgroups. **(A)** Age; The red curve represents the population over 60 years old and the green curve represents the population under 60 years old **(B)** Sex; The red curve represents males, and the green curve represents females. PIV, pan-immune inflammation value; CHD, coronary heart disease.

It was employed to investigate the curve relationship between InPIV and CHD. Our results uncover a non-linear positive link between InPIV and CHD (*P* for overall <0.001, *P* for non-linear = 0.009). At first, as InPIV rose, the incidence of CHD slowly increased. However, the incidence of CHD increased significantly when it exceeded a certain threshold (Figure 2). Interestingly, this positive correlation seems to be more pronounced in the elderly population (Figure 3A). This positive correlation has also been noted in both male and female participants (Figures 3B).

### Sensitivity analysis

Studies have indicated that outcomes from weighted analyses and unweighted analyses may occasionally differ. Therefore, we employ unweighted approaches to confirm the reliability of our findings. As a continuous variable, a positive correlation was showed between PIV and CHD, with an OR of 1.12 (95%CI: 1.04–1.21) in Model II. Compared to the Q1 quartile of InPIV, the Q4 quartile exhibits the greatest risk of CHD (OR = 1.24, 95% CI: 1.06–1.45) in Model II (Table 3).

**Table 3:** Unweighted logistic regression analysis on the correlation between PIV and CHD in sensitivity analysis.

	Non-adjusted model		Model I		Model II	
	OR [95% CI]	*P* value	OR [95% CI]	*P* value	OR [95% CI]	*P* value
Continuous PIV	1.44 (1.34, 1.54)	<0.001***	1.16 (1.08, 1.25)	<0.001***	1.12 (1.04, 1.21)	0.004**
Q1	Reference	–	Reference	–	Reference	–
Q2	1.27 (1.09, 1.47)	0.002**	1.12 (0.96, 1.32)	0.15	1.14 (0.97, 1.34)	0.12
Q3	1.45 (1.25, 1.68)	<0.001***	1.20 (1.03, 1.40)	0.02*	1.18 (1.00, 1.38)	0.05
Q4	1.91 (1.66, 2.20)	<0.001***	1.33 (1.14, 1.54)	<0.001***	1.24 (1.06, 1.45)	0.01*

Data are presented as OR (95% CI). PIV, pan-immune-inflammation value; CHD, coronary heart disease; Model I adjusted for age, sex and race/ethnicity. Model II adjusted for age, sex, race/ethnicity, education levels, BMI, smoking, drinking, TC, HDL-C, uric acid, eGFR, hypertension and DM.

* *P* value <0.05.

** *P* value <0.01.

*** *P* value <0.001.

### Subgroups analysis

To further elucidate the link between PIV and CHD, subgroup analysis was utilized to investigate the potential link between PIV and CHD in different subgroups based on age, sex, BMI, race, education levels, smoking, drinking, hypertension and DM. However, there were no notable differences found in the impact of PIV on CHD within the other subgroups. Interestingly, this relationship is particularly significant in elderly patients aged 60 and above (Table 4).

**Table 4:** Subgroup analysis for the correlation between PIV and CHD.

Variable name	Non-CHD	CHD	*p* value	*p* for interaction
Age				0.42
≤60	Ref	1.18 (0.94, 1.48)	0.15	
>60	Ref	1.32 (1.17, 1.49)	<0.001	
Sex				0.19
Male	Ref	1.34 (1.19, 1.52)	<0.001	
Female	Ref	1.54 (1.30, 1.83)	<0.001	
BMI				0.08
<25	Ref	1.70 (1.34, 2.16)	<0.001	
25–30	Ref	1.23 (1.04, 1.46)	0.01	
>30	Ref	1.35 (1.15, 1.58)	<0.001	
Race				0.64
White	Ref	1.38 (1.22, 1.55)	<0.001	
Black	Ref	1.17 (0.94, 1.46)	0.16	
Mexican American	Ref	1.29 (0.95, 1.74)	0.10	
Others	Ref	1.26 (0.91, 1.75)	0.17	
Education levels				0.33
Less than high school	Ref	1.25 (1.06, 1.46)	0.01	
High school or Equivalent	Ref	1.42 (1.18, 1.71)	<0.001	
College or above	Ref	1.47 (1.27, 1.70)	<0.001	
Smoking				0.34
No	Ref	1.44 (1.19, 1.73)	<0.001	
Yes	Ref	1.29 (1.15, 1.45)	<0.001	
Drinking				0.39
No	Ref	1.48 (1.27, 1.72)	<0.001	
Yes	Ref	1.36 (1.21, 1.54)	<0.001	
Hypertension				0.61
No	Ref	1.34 (1.05, 1.71)	0.02	
Yes	Ref	1.25 (1.12, 1.39)	<0.001	
DM				0.73
No	Ref	1.35 (1.19, 1.54)	<0.001	
Yes	Ref	1.30 (1.11, 1.53)	0.001	

PIV, pan-immune-inflammation value; BMI, body mass index; CHD, coronary heart disease; DM, diabetes mellitus.

### ROC curve analysis

**Figure 4:**
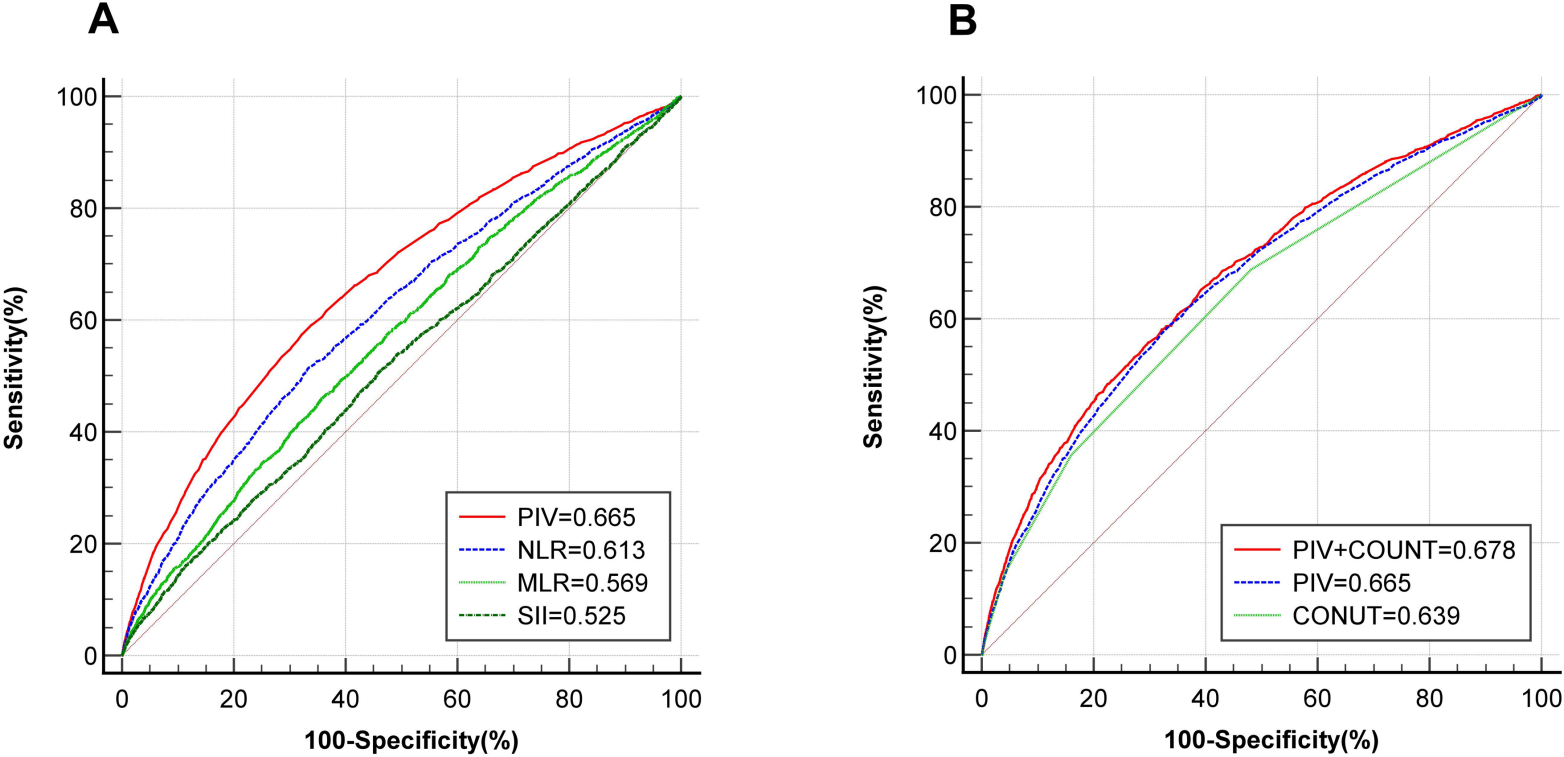
ROC curve reveals the diagnostic value of PIV for CHD. **(A)** Comparison of the predictive value of PIV(red), NLR(blue), MLR(light green) and SII(deep green) for CHD; **(B)** The predictive value of PIV(blue), COUNT score(light green) and PIV combined with COUNT score(red) for CHD. ROC, receiver operator characteristic; PIV, pan-immune inflammation value; NLR, neutrophil-to-lymphocyte ratio; MLR, monocyte-to-lymphocyte ratio; SII, systemic immune inflammation index; COUNT, controlling nutritional status; CHD, coronary heart disease.

ROC curve analysis shows that PIV has better predictive value for CHD than NLR, MLR and SII (PIV vs. NLR vs. MLR vs. SII: 0.665 vs. 0.613 vs. 0.569 vs. 0.525). The ROC curve cut-off value of PIV is 324. The sensitivity is 57.47% and the specificity is 67.81%. Due to the close correlation between inflammation and malnutrition, ROC curve analysis also found that PIV combined with COUNT score has greater predictive value for CHD (PIV + COUNT vs. PIV vs. COUNT: 0.678 vs. 0.665 vs. 0.639) (Figure 4).

## Discussion

This research demonstrates that a positive relationship existed between PIV and CHD, especially in elderly people. The predictive value of PIV for CHD is superior to NLR, MLR and SII. The combination of PIV and COUNT score has better predictive value for CHD.

Chronic inflammation is vital in the development of atherosclerosis. In the early stage of atherosclerosis, neutrophils can induce endothelial dysfunction, promote the formation of foam cells and inflammatory response. In addition, neutrophils also participate in plaque vascular injury, necrotic nucleus formation, and thinning of fibrous caps, promoting unstable plaque rupture and activating platelets, leading to secondary thrombosis (29, 30). In addition, the chemotaxis, adhesion and migration of monocytes are also involved in the regulation of atherosclerosis inflammation (31). platelets can combine with a variety of cells and factors, participate in cascade inflammatory reaction, and promote the formation of atherosclerosis (32). Lymphocytes participate in the immune regulation of atherosclerosis, and maintain the balance between proinflammatory and anti-inflammatory responses (33). The interaction of these cells may promote the progression of atherosclerosis.

In recent years, researchers have developed various inflammatory indices, including NLR and MLR, which are associated with prognosis in various diseases. In addition, the relationship between these indicators and CHD is constantly being confirmed (34, 35). Recently, some scholars found that PIV integrates four types of blood cell counts, which can better reflect the inflammatory and immune condition, and has shown good value in various diseases. Furthermore, studies have found that PIV is associated with slow coronary flow and adverse cardiovascular events (36, 37). Similarly, our findings indicate that higher levels of PIV are linked to a greater risk of CHD, and this relationship is superior to other inflammatory markers. In clinical practice, PIV integrates four types of blood cells, which can better reflect systemic inflammation. Therefore, it has good evaluation value for patients’ disease status as a novel inflammatory marker, especially in CHD. In addition, several studies have discovered that patients with CHD are often older and mostly suffer from malnutrition. The COUNT score is commonly used for predicting the prognosis of inflammatory diseases or chronic wasting diseases, with good predictive performance and convenient use (38, 39). There is an inseparable relationship between malnutrition and inflammation. Inflammation can induce malnutrition, which in turn exacerbates inflammation (40). Our study also suggests that PIV combined with COUNT score has better predictive value for CHD. Our findings may provide help for the management of CHD.

It is noteworthy that the positive link between elderly people was significant. Aging is a chronic inflammatory process, and chronic inflammation can further exacerbate aging in turn. Therefore, It is relatively easy to grasp that inflammation may play a more important role in elderly patients. Additionally, malnutrition prevalence is elevated among older adults, and we also found that PIV combined with COUNT score has greater predictive value for CHD.

This study provides important references for the management of CHD patients. The NHANES database, which is extensive and international, offers robust support for the risk assessment of CHD. Compared with other common clinical inflammatory indices, PIV can comprehensively reflect the systemic inflammatory status, which provides better help for the health of Americans. However, our research also has some limitations. First, we cannot determine the causal relationship. Second, we have not monitored the dynamic changes in hematological indices. Third, the results of this study are mainly applicable to the American population. In addition, the diagnostic criteria for CHD is based on a history of CHD. Furthermore, CHD is also classified into stable angina and acute coronary syndrome according to severity in clinical practice. We did not explore the correlation between PIV and the severity of CHD in this study. Finally, there might still be other confounding elements that could affect the results, including medication use, dietary habits, and physical activity. In the future, we will further explore the relationship between PIV and CHD by combining clinical cases.

## Conclusion

A positive relationship was observed between PIV and CHD, especially in the elderly. The combination of PIV and COUNT score has better diagnostic value for CHD.

## Data Availability

Publicly available datasets were analyzed in this study. This data can be found here: https://www.cdc.gov/nchs/nhanes/.
