# Supplementary material for: Association between pan-immune-inflammation value and coronary heart disease in elderly population: a cross-sectional study
Source: Front Cardiovasc Med. 2025 Feb 10;12:1538643. doi: 10.3389/fcvm.2025.1538643 (PMC11847815; doi:10.3389/fcvm.2025.1538643)
Supplement: Supplementary file 1 [file Table1.docx]

**Table 1. The characteristics of the population according to the quartile of the PIV.**

| **Variables** | **Overall** | **Q1** | **Q2** | **Q3** | **Q4** | ***P* value** |
| --- | --- | --- | --- | --- | --- | --- |
| **Age, %** | 47.21±0.19 | 46.06±0.26 | 46.57±0.23 | 47.24±0.26 | 48.74±0.26 | <0.001*** |
| **Sex, %** |  |  |  |  |  | 0.8 |
| Female | 50.54(48.64,52.44) | 51.10(49.90,52.31) | 50.33(49.13,51.53) | 50.44(49.34,51.53) | 50.36(49.24,51.48) |  |
| Male | 49.46(47.66,51.27) | 48.90(47.69,50.10) | 49.67(48.47,50.87) | 49.56(48.47,50.66) | 49.64(48.52,50.76) |  |
| **Race/ethnicity, %** |  |  |  |  |  | <0.001*** |
| White | 70.05(65.84,74.27) | 58.75(56.17,61.33) | 70.47(68.36,72.59) | 72.82(70.75,74.89) | 76.46(74.48,78.43) |  |
| Black | 10.22( 9.34,11.11) | 19.04(17.16,20.93) | 9.60( 8.54,10.67) | 7.60( 6.73, 8.47) | 5.96( 5.22, 6.69) |  |
| Mexican | 8.01( 7.04, 8.98) | 8.31(7.17,9.45) | 8.29(7.11,9.48) | 8.15(7.02,9.29) | 7.34(6.20,8.49) |  |
| Others | 11.71(10.75,12.67) | 13.90(12.61,15.18) | 11.63(10.39,12.87) | 11.43(10.25,12.61) | 10.24( 9.02,11.47) |  |
| **Education levels, %** |  |  |  |  |  | <0.001*** |
| Less than high school | 16.49(15.52,17.45) | 16.70(15.59,17.80) | 16.06(14.89,17.22) | 15.54(14.40,16.67) | 17.65(16.49,18.81) |  |
| High school or equivalent | 23.98(22.66,25.29) | 21.54(20.29,22.80) | 22.64(21.45,23.83) | 24.47(23.29,25.65) | 26.83(25.58,28.08) |  |
| College or above | 59.54(57.01,62.06) | 61.76(59.98,63.54) | 61.30(59.61,62.99) | 59.99(58.27,61.71) | 55.52(53.82,57.23) |  |
| **BMI, kg/m2** | 28.77±0.07 | 27.67±0.09 | 28.34±0.10 | 29.14±0.10 | 29.75±0.11 | <0.001*** |
| **HbA1c, %** | 5.57±0.01 | 5.53±0.01 | 5.53±0.01 | 5.57±0.01 | 5.63±0.01 | <0.001*** |
| **TC, mmol/L** | 5.08±0.01 | 5.03±0.02 | 5.10±0.01 | 5.10±0.02 | 5.07±0.01 | 0.001** |
| **HDL-C, mmol/L** | 1.37±0.00 | 1.42±0.01 | 1.39±0.01 | 1.35±0.01 | 1.34±0.01 | <0.001*** |
| **Uric acid, umol/L** | 322.85±0.63 | 314.38±1.14 | 320.85±1.14 | 323.71±1.01 | 331.07±1.18 | <0.001*** |
| **eGFR, mL/min/1.73 m^2^** | 93.79±0.26 | 95.76±0.35 | 94.37±0.31 | 93.80±0.35 | 91.58±0.36 | <0.001*** |
| **COUNT score** | 0.70±0.01 | 0.68±0.01 | 0.64±0.01 | 0.65±0.01 | 0.81±0.01 | <0.001*** |
| **Smoking, %** |  |  |  |  |  | <0.001*** |
| No | 53.42(51.50,55.35) | 59.28(57.88,60.69) | 56.16(54.61,57.71) | 52.31(50.94,53.68) | 46.94(45.45,48.44) |  |
| Yes | 46.58(44.47,48.68) | 40.72(39.31,42.12) | 43.84(42.29,45.39) | 47.69(46.32,49.06) | 53.06(51.56,54.55) |  |
| **Drinking, %** |  |  |  |  |  | <0.001*** |
| No | 25.60(23.90,27.30) | 26.60(24.95,28.26) | 24.33(22.64,26.02) | 24.63(23.04,26.22) | 26.94(25.42,28.45) |  |
| Yes | 74.40(71.60,77.20) | 73.40(71.74,75.05) | 75.67(73.98,77.36) | 75.37(73.78,76.96) | 73.06(71.55,74.58) |  |
| **Hypertension, %** |  |  |  |  |  | <0.001*** |
| No | 62.90(60.57,65.24) | 67.69(66.42,68.97) | 65.87(64.42,67.32) | 62.22(60.82,63.62) | 56.69(55.31,58.07) |  |
| Yes | 37.10(35.47,38.72) | 32.31(31.03,33.58) | 34.13(32.68,35.58) | 37.78(36.38,39.18) | 43.31(41.93,44.69) |  |
| **DM, %** |  |  |  |  |  | <0.001*** |
| No | 87.30(84.13,90.47) | 88.86(88.09,89.63) | 88.89(88.20,89.57) | 87.00(86.09,87.91) | 84.77(83.88,85.66) |  |
| Yes | 12.70(12.04,13.35) | 11.14(10.37,11.91) | 11.11(10.43,11.80) | 13.00(12.09,13.91) | 15.23(14.34,16.12) |  |
| **CHD, %** |  |  |  |  |  | <0.001*** |
| No | 96.49(93.06,99.93) | 97.43(97.04,97.83) | 96.77(96.34,97.20) | 96.46(95.95,96.97) | 95.47(94.98,95.97) |  |
| Yes | 3.51( 3.20, 3.81) | 2.57(2.17,2.96) | 3.23(2.80,3.66) | 3.54(3.03,4.05) | 4.53(4.03,5.02) |  |

Continuous data were presented as the mean±SEM, category data were presented as the proportion and 95% confidence interval. SEM, Standard Error of the Mean; PIV, pan-immune-inflammation value; BMI, body mass index; HbA1c, glycosylated hemoglobin; TC, total cholesterol; HDL-C, high-density lipoprotein cholesterol; eGFR estimated glomerular filtration rate; DM, diabetes mellitus; CHD, coronary heart disease; *** P value<0.001, ** P value<0.01, * P value<0.05.
