# Supplementary material for: Association between pan-immune-inflammation value and coronary heart disease in elderly population: a cross-sectional study
Source: Front Cardiovasc Med. 2025 Feb 10;12:1538643. doi: 10.3389/fcvm.2025.1538643 (PMC11847815; doi:10.3389/fcvm.2025.1538643)
Supplement: Supplementary file 2 [file Table2.pdf]

Table S2. The calculation of COUNT scores.

Variable				
**Albumin(g/dL)**	≥3.5	3-3.4	2.5-2.9	<2.5
Albumin score	0	2	4	6
**TC(mg/dL)**	≥180	140-79	100-139	<100
TC score	0	1	2	3
**lymphocytes (count/mL)**	≥1600	1200-1599	800-1199	<800
Lymphocytes score	0	1	2	3

CONUT, Controlling nutritional status; TC, total cholesterol
